# On measuring selection in cancer from subclonal mutation frequencies

**DOI:** 10.1371/journal.pcbi.1007368

**Published:** 2019-09-26

**Authors:** Ivana Bozic, Chay Paterson, Bartlomiej Waclaw

**Affiliations:** 1 Department of Applied Mathematics, University of Washington, Seattle, Washington, United States of America; 2 School of Physics and Astronomy, University of Edinburgh, Edinburgh, United Kingdom; University of Minnesota, UNITED STATES

## Abstract

Recently available cancer sequencing data have revealed a complex view of the cancer genome containing a multitude of mutations, including drivers responsible for cancer progression and neutral passengers. Measuring selection in cancer and distinguishing drivers from passengers have important implications for development of novel treatment strategies. It has recently been argued that a third of cancers are evolving neutrally, as their mutational frequency spectrum follows a 1/*f* power law expected from neutral evolution in a particular intermediate frequency range. We study a stochastic model of cancer evolution and derive a formula for the probability distribution of the cancer cell frequency of a subclonal driver, demonstrating that driver frequency is biased towards 0 and 1. We show that it is difficult to capture a driver mutation at an intermediate frequency, and thus the calling of neutrality due to a lack of such driver will significantly overestimate the number of neutrally evolving tumors. Our approach provides quantification of the validity of the 1/*f* statistic across the entire range of relevant parameter values. We also show that our conclusions remain valid for non-exponential models: spatial 3d model and sigmoidal growth, relevant for early- and late stages of cancer growth.

## Author summary

Darwinian evolution in cancer is responsible for the emergence of malignant traits in initially benign tumors. As tumor cells divide, they accumulate new mutations and while most of them are “passengers” which do not confer any selective growth advantage, “driver” mutations endow cells with traits that contribute to cancer spread. Identifying driver mutations that are under selection in cancer can point to new targets for cancer therapeutics and open new avenues for personalized cancer treatment. It has recently been argued that the presence or absence of selection in cancer can be deduced from deviation of mutant allele frequencies from 1/*f* power law in an intermediate frequency range. Using a stochastic mathematical model of cancer evolution we derive a formula for the frequency of a subclonal driver and show that frequencies of cancer drivers are biased towards 0 and 1; thus most mutations will inevitably appear to be either neutral (frequency ≈ 0) or clonal (frequency ≈ 1) despite very different levels of selection. Consequently, the proposed 1/*f* statistic will significantly overestimate the number of cancers deemed to be evolving neutrally. Our work quantifies the validity of the proposed neutral evolution statistic across the entire range of relevant parameter values.

## Introduction

Darwinian evolution in cancer has been the subject of intense research in the past decade. In particular, the problem of distinguishing driver mutations that carry a selective advantage from passenger mutations, and their role in shaping intra-tumor genetic heterogeneity has come to the fore [1–5]. Determining which mutations in cancer are drivers and which are passengers is one of the most pressing questions in cancer genomics, as identification of new driver mutations can contribute to development of new targeted therapeutics [6,7] and personalized medicine [8]. Numerous methods for classifying driver and passenger mutations and measuring selection in cancer have been developed, including those that identify driver genes based on how frequently they are mutated [2], specific mutation patterns [9,10], and dN/dS ratios [1,11]. These methods can reliably identify driver genes mutated in a high proportion of tumors of a given type (>20%); using such methods to find less common drivers would require a large number of cancer samples [12], and drivers unique to a single or a small number of patients could still be missed.

Several recent papers attempt to measure the magnitude of selection operating during cancer evolution using the frequency distribution of subclonal mutations in an individual patient’s cancer. In a seminal paper, Williams et al. used mutant allele frequencies to conclude that a significant fraction (~1/3) of cancers evolve neutrally [13]. Subsequent studies focused on quantifying the strength of selection and distinguishing it from “effectively neutral” cancer evolution [14,15]. These works [13,15] are based upon the assumption that drivers that arose after cancer initiation will be present at a macroscopic but clearly subclonal frequency (i.e. “detectable”), which will make the cumulative mutant allele frequency look different to the 1/*f* power law expected from neutral evolution. Here we use a branching process model of cancer evolution to derive a formula for the probability of detection of a subclonal driver, and test the validity of the proposed 1/*f* statistic across all relevant parameter combinations.

## Results

**Fig 1:**
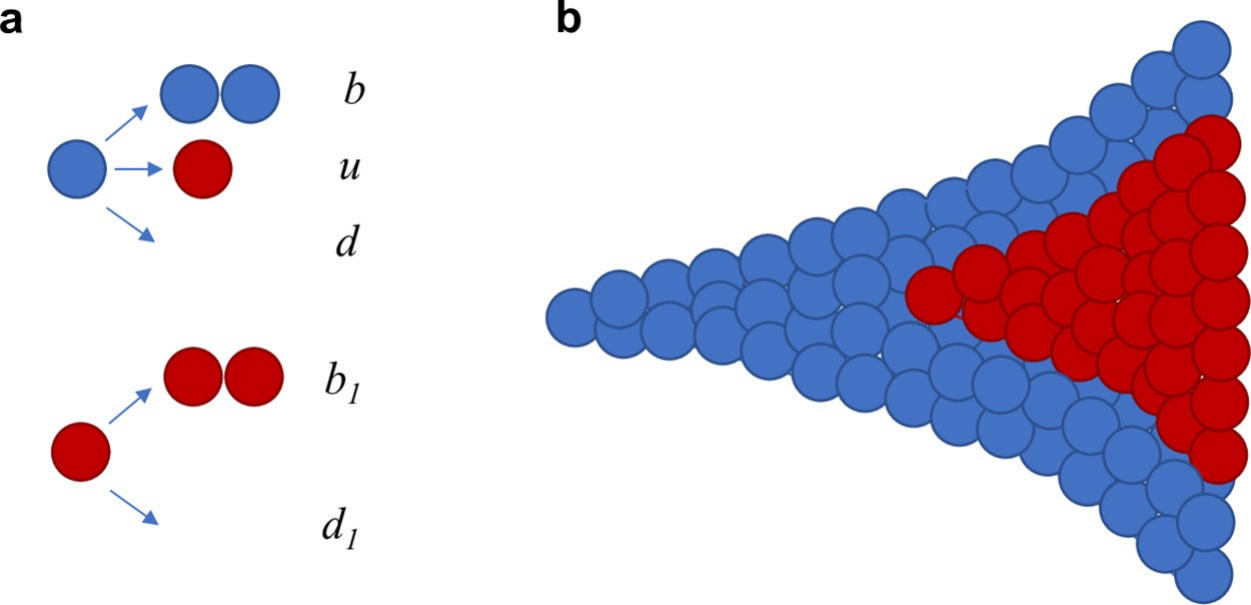
Schematic representation of the stochastic model of tumor evolution. **a**, Transformed cells (blue) divide with rate *b*, obtain an additional driver mutation with rate *u*, and die with rate *d*. Cells with the additional driver (red) divide with rate *b*_1_ and die with rate *d*_1_. The ratio of net growth rates of cells with and without the driver, *c* = (*b*_1_-*d*_1_)/(*b*-*d*) is greater than 1. **b**, Growth begins with a single parental cell. We are interested in the fraction of cells with the driver as a function of the total number of tumor cells *M*.

We consider a two-type stochastic model of cancer evolution (Fig 1a). In the model, cancer is initiated by a single transformed cell. Progeny of this cell follow a branching process with birth rate *b* and death rate *d*. We set *r* = *b* − *d* > 0, so the population grows if it survives initial stochastic fluctuations. In addition, cancer cells can obtain a new driver mutation with rate *u* (see Materials and methods). Cells with the driver mutation replicate with rate *b*_1_ and die with rate *d*_1_ smaller than *b*_1_ (Fig 1a and 1b). The subpopulation of driver-carrying cells has therefore a net growth rate *r*_1_ = *b*_1_ − *d*_1_, and we assume that *r*_1_ > *r* so that the additional driver increases the net growth rate by the factor *c* = *r*_1_/*r* > 1. We define *g* = *c* − 1 as the relative increase in the growth rate due to the driver.

We are interested in the frequency of cancer cells that carry the driver mutation. In a neutral process (*g* = 0), mutation frequency stabilizes and remains approximately constant at large times [16]. For *g* > 0 (driver with a selective advantage), the frequency of cells with the driver increases from ≈0 to ≈100% during tumor expansion. We denote by *F*[*α*] the probability that subclonal driver frequency *f* is smaller than *α*. We show (Materials and methods) that this cumulative frequency distribution reads

$$F\left[\alpha \right]\approx \overset{0}{\underset{M}{\int }}\frac{cu}{b_{1}}\text{exp}\left(-\frac{cu}{b_{1}}X\right)\left[1-\text{exp}\left(-\frac{cr}{b_{1}}\frac{\alpha }{{\left(1-\alpha \right)}^{c}}X^{c}M^{1-c}\right)\right]dX$$ (1)

where denotes *M* is number of cells in the tumor. Formula (1) is in excellent agreement with exact computer simulations of the branching process (Materials and methods). Recently, similar two-type processes were studied by Kessler and Levine [17] and Cheek and Antal [18], who derived generating functions [18] and probability distributions for the size of the mutant population in the case of small mutant frequencies [17] in the general asymmetric (different fitness of wildtype and mutant cells) Luria-Delbruck model with death. In contrast, our formula (1) describes the probability distribution for a single driver subclone that has reached non-negligible frequencies.

We note that Eq (1) concerns the cancer cell frequency of a subclonal driver, and not the variant allele frequency obtained from genomic analysis. As noted recently [19], variant allele frequency does not automatically indicate a certain cancer cell frequency (due to contamination with normal cells and variable ploidy), and using allele frequencies instead of cancer cell frequencies to detect selection can be an additional source of bias.

We assume that a subclonal driver mutation can be detected, and able to skew the 1/*f* power law expected from neutral evolution, when its cancer cell frequency is between 20% and 80%. This range is much wider than the range 24% to 48% used in Williams et al. [13] (mutant allele frequency range 12% to 24%). Thus, the probability that a driver can be detected is given by

$$P_{det}=F[0.8]-F[0.2]$$ (2)

**Fig 2:**
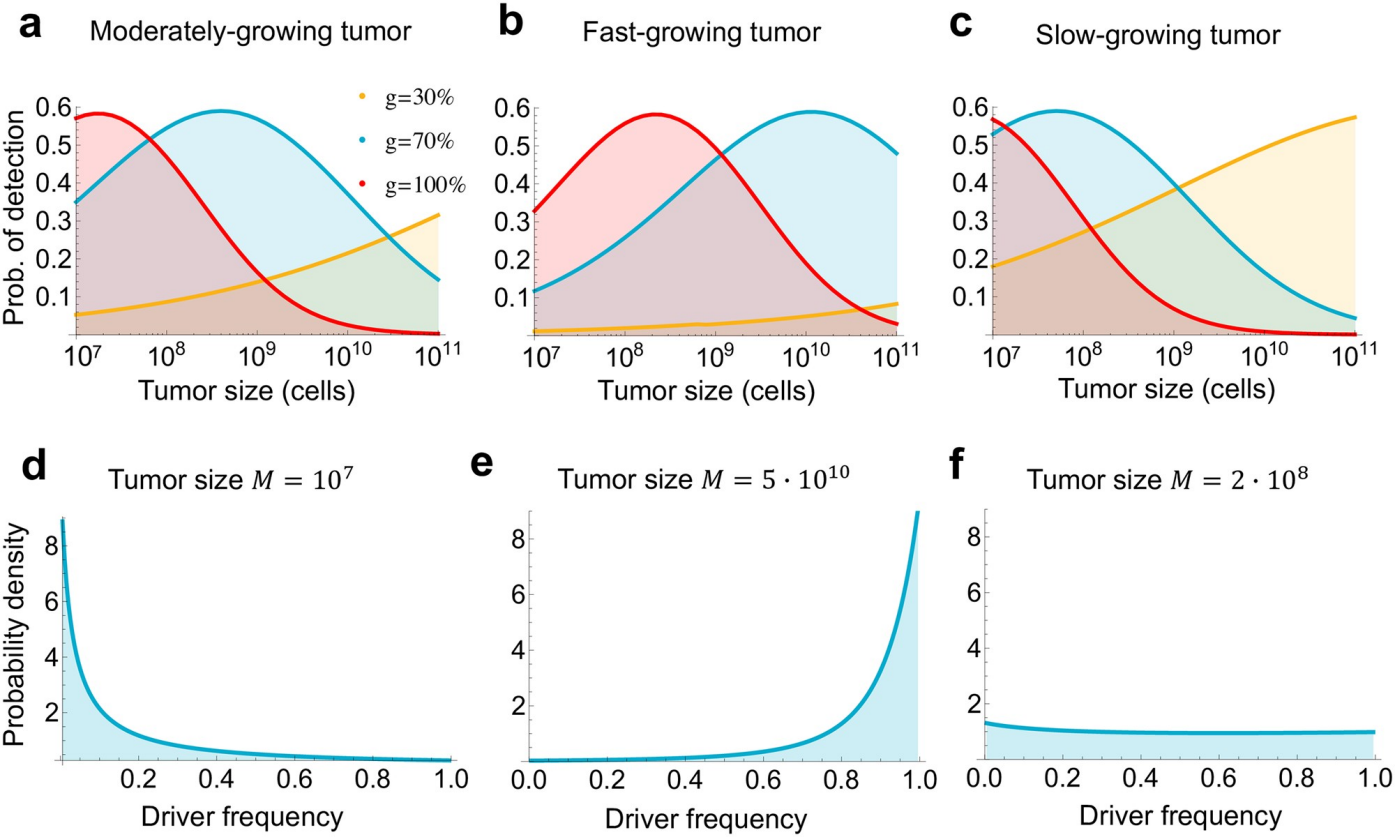
Cancer cell frequency of a subclonal driver is biased towards 0 and 1. **a, b, c**, Probability that a subclonal driver is in the detectable range (0.2 ≤ *f*_*sub*_ ≤ 0.8), and thus able to skew the distribution of mutational cancer cell frequencies expected from neutral evolution, for three parameter regimes. For each parameter regime, we depict three levels of selection: moderate selection (driver increases net growth rate by *g* = 30%), strong selection (*g* = 70%), and very strong selection (*g* = 100%). Parameter values for **a**, moderately growing tumor [20]: *b* = 0.14, *r* = 0.01; **b**, fast growing tumor [36]: *b* = 0.25, *r* = 0.07; **c**, slow-growing tumor [21]: *b* = 0.33, *r* = 0.0013. Driver mutation rate [21] *u* = 10^−5^. All rates are per day and *b* = *b*_1_. **d, e, f**, Probability density for the frequency of a subclonal driver that increases the net growth rate by 70%, in a moderately growing tumor (**a**). **d**, Driver frequency is biased towards 0 when tumor size is small. **e**, When tumor size is large, driver frequency is biased towards 1. **f**, When detection is most likely (at intermediate size), driver frequency distribution is almost flat.

For moderate levels of selection, e.g., when the additional driver mutation increases the growth rate by *g* = 30%, the probability that the driver mutation is in the detectable range ([0.2,0.8]) is <15% for population sizes up to *M* = 10^9^ cells, and remains below one third for *M* ≤ 10^11^ cells (Fig 2a). For other cases considered here (70% and 100% increase in the net growth rate), the chance of detecting the subclonal driver is always <60% and—for a broad range of tumor sizes—less than 30%. The parameters used in Fig 2a are from Bozic et al. [20], and are typical for a moderately aggressive cancer (net growth rate 0.01/day). We show that the situation is qualitatively similar for faster and slower growing cancers in Fig 2b and 2c. In summary, for moderate levels of selection (*g* = 30%), the chance of detecting a subclonal driver is small for almost any tumor size, and very strong selection (*g* = 100%) will be detectable only in small cancers. Strong selection (*g* = 70%) will be detectable at intermediate-size, moderately growing tumors; large, fast-growing tumors; or small, slow-growing tumors. Most notably, for all parameter values, even in the parameter regimes where the probability of detecting the subclonal driver is the highest, it is still below 60% (Fig 2a–2c).

Our results suggest that detecting deviation from neutral evolution is challenging, as there is a significant chance that a subclonal driver will not be in the detectable range. The reason is that the frequency of cells with the new driver is biased toward 0 and 1. When the tumor is small, the fraction of driver-carrying cells is very close to zero, as there has not yet been enough time for the fitter subpopulation to expand (Fig 2d). In contrast, for large tumors, driver-carrying subpopulation has already expanded and completely dominates the population, so its frequency is close to 100% (Fig 2e). Interestingly, for sizes at which the chance of detecting the subclonal driver is highest (close to 60%), the frequency distribution is almost flat (Fig 2f).

**Fig 3:**
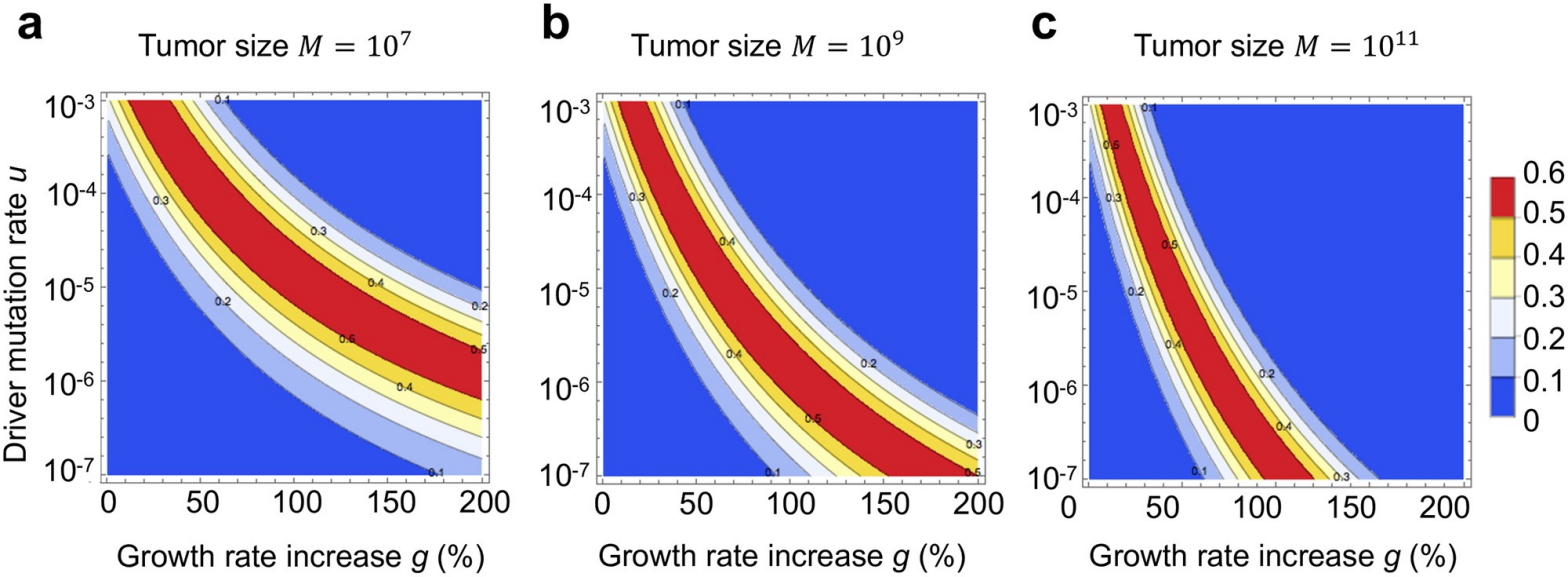
Probability of detection of a subclonal driver for a wide range of driver mutation rates and growth rate advantages is always below 60%. Contour plots depict the probability that a subclonal driver is in the detectable range (0.2 ≤ *f*_*sub*_ ≤ 0.8) and thus able to skew the distribution of mutation frequencies expected from neutral evolution, for **a**, small; **b**, intermediate; and **c**, large tumor size. Parameter values for moderately growing tumor *b* = *b*_1_ = 0.14, *r* = 0.01. All rates are per day.

In Fig 2 we used a previously estimated driver mutation rate *u* = 10^−5^ per day [21]. To explore the effect of a higher or lower driver mutation rate on our conclusions, we first used recently published genomic data to determine an upper bound (10^−3^) and a lower bound (10^−7^ per day) on the driver mutation rate (Materials and methods). We next performed a numerical grid search on the space of all parameters (driver mutation rate *u*, relative growth rate advantage of a driver *g*, net growth rate of tumor cells *r*, division rate of cells with the driver *b*_1_, and final number of tumor cells *M*). A wide range of values is taken for each parameter, including driver mutation rate *u* between 10^−7^ and 10^−3^ per day, and growth advantage of a subclonal driver *g* between 1% and 200% (see Materials and methods for more details). The grid search demonstrated that the probability of detection of a subclonal driver is always below 60%, and that subclonal driver frequency is biased towards 0 and 1 across the entire range of reasonable parameter values of the carcinogenic process (Materials and methods, Fig 3). The intuitive reason behind this result is that the probability density function for subclonal driver frequency is convex across this entire parameter range (examples in Fig 2d, 2e and 2f).

The well-mixed model discussed so far does not include spatial constrains experienced by solid tumors. We thus extended our analysis to a similar two-type process in three-dimensional space, using a lattice-based computer model [5]. We considered a version of the model in which cells that occupy points of a 3d lattice replicate to empty neighboring sites, die, and mutate, but do not migrate [5]. Normal cells replicate and die with rates *b*, *d*, whereas mutant cells replicate with rate *b* but die with rate *d*_1_. Similarly as before, the ratio of net growth rates of cells with and without the driver is given by *c* = 1 + *g* = (*b* − *d*_1_) / (*b − d*) >1. To simulate a large number of tumors for realistic sizes, we scaled the parameters of the model so that 1 site corresponded to 100 cells, and mutation probability was 100x larger.

S1 Fig shows the probability of detection of a subclonal driver as a function of tumor size for medium, fast and slow tumor growth, and for three levels of driver potency (*g* = 30%, 70% and 100% increase in net growth rate *b*-*d*). These results demonstrate that our main conclusion that many drivers elude detection remains true in the 3d model. In particular, out of 36 parameter combinations evaluated, the probability of detection of a subclonal driver was below 60% in 33 out of 36 cases, and in the remaining 3 cases the probability was 63%, 67% and 68%. The probability of detection was 40% or below for one half of the parameter sets. Of note, we observe that in the 3d model there is a higher chance of detection of moderate drivers (g = 30%) compared to the well-mixed model, and the chance of detection peaks at lower tumor sizes compared to the well-mixed case.

The growth of tumor populations is complex and may change throughout the carcinogenic process. For example, initial growth may be slower than exponential due to tissue constraints and nutrient availability, exponential-like after angiogenesis, and may slow down again when the tumor is very large. To model this more complex scenario, we use the 3d model described above to model the first, avascular stage, before tumor reaches 10^6^ cells [22,23]. As 10^6^ cells is typically at the lower end of tumor detectability, it is unlikely that drivers will be detected at this stage. To model the later stages of tumor growth (exponential and slow-down), we employ the following system of differential equations:

$$\frac{dx}{dt}=rx(1-\frac{x+y}{K})$$

$$\frac{dy}{dt}=cry(1-\frac{x+y}{K})$$

Here *x* is the size of the type-0 and *y* is the size of the type-1 (driver) population, *c* = 1+g >1 is the ratio of their initial growth rates and *K* is the carrying capacity of the tumor. We use a deterministic model because both wild-type and driver populations are likely to be large at the end of the spatial phase. To combine the 3d and the above model, we record the sizes of the type-0 and type-1 populations obtained from the 3d simulation when total population size reaches 10^6^ cells, and use them as initial conditions for the system described above, which we solve numerically. We show results for probability of driver detection in this sigmoidal model in S2 Fig.

In the sigmoidal growth model, in contrast to the well-mixed and 3d models, driver fraction does not approach 1 for large tumors. Instead, it reaches a stable frequency that depends on the initial driver fraction and model parameters. For all parameter combinations we evaluated, the chance that this final driver frequency is between 0.2 and 0.8 is below 63%. We note that the sigmoidal model increases the chance of detection of moderate drivers (g = 30%) compared to the well-mixed model, but decreases the chance of detection of strong drivers (g = 70% and g = 100%), as they will expand to the carrying capacity quickly and not leave much room for the type-0 population. In this model, selection will in general be more detectable for slower growing compared to faster-growing tumors. In sum, our results imply that in the sigmoidal model, low to moderate levels of selection will be most detectable at the final (close to carrying capacity) stage, whereas strong drivers will be most detectable at the beginning of the exponential-like phase. We note, however, that sigmoidal growth models may produce different behaviors depending on the specifics of the competition between the two populations—for example not only initial growth rates but also the levels of growth inhibition may differ between populations, and cell turnover while the population is at carrying capacity may lead to competitive exclusion of the less fit population.

Finally, we also considered how the appearance of a second driver within the first driver population would influence probability of detection of either driver for realistic parameter values in the well-mixed model. To that end, we derived the expression for the probability that the second driver will be in the detectable range, assuming the first driver is not detectable,

$$P_{2}\approx \int_{0.8}^{1}f_{1}(\alpha_{1})(F_{2}(0.8/\alpha_{1})-F_{2}(0.2/\alpha_{1}))d\alpha_{1}$$

Here *f*_1_ is the probability density of the first driver frequency (Eq (7) in Materials and methods) and *F*_2_ is the cumulative probability for the frequency of the second driver in the first driver population (Eq (1) with appropriate parameter values).

For the same parameters as in Fig 2, and assuming that second driver increases the net growth rate by the same absolute amount as the first, we show that the probability that driver frequency is in the range [0.2,0.8] is always below 69% (S3 Fig). In addition to this upper bound, we also calculate the average probability of driver detection across all parameter values evaluated in S3 Fig. The average detection probability is 43% when one includes driver mutations in cancer cell frequency (CCF) range [0.2, 0.8]. The average driver detection probability for the CCF range [0.24, 0.48] from the original Williams et al. study [13] is 18%.

## Discussion

In sum, the fact that no subclonal driver is present at intermediate frequencies cannot be taken as proof of neutral evolution. It can simply be a consequence of population dynamics which creates only a short window during which the driver mutation can be detected but has not yet dominated the population.

Tarabichi et al. [24] and McDonald and colleagues [25] simulated tumor evolution in which they explicitly include selection, and showed that, even in models with selection, mutant allele frequency can exhibit the 1/*f* power law behavior, resulting in incorrect calling of neutrality. In response, Williams and colleagues [26,27] argue that the example simulations from Tarabichi et al. [24] and McDonald et al. [25] that were incorrectly classified as neutral use extreme parameter values or correspond to either strong and early selection (a driver mutant quickly sweeps to fixation), or weak and late selection (driver mutants unable to reach detectable frequencies). In contrast, we show here that, for almost any driver mutation rate and selection strength, whenever we look at the mutant frequency spectrum of a tumor, it is likely either too early and the driver is present at a very low frequency, or it is already too late, and the driver is present in almost all cells of the tumor. Importantly, even if we manage to obtain the mutant frequency spectrum during the optimal window for detection, there is still significant chance (close to half) that the subclonal driver will not be in the detectable range. Thus, even though multiple studies [13,28] (including ours [16]) have confirmed that the experimental allele frequency spectrum of many cancers agrees with the spectrum of a neutral model in a certain frequency range, we argue that this agreement should not be taken as evidence of neutral evolution.

Simulations of branching processes of cancer evolution for realistic tumor sizes and parameter values are computationally expensive. To circumvent that, studies often use small population sizes, death rate of cancer cells much smaller than the birth rate, and only examine a small set of different parameter values. In contrast, our mathematical results (formula 2) can be quickly evaluated for realistic parameter values, including all biologically plausible values of selection, mutation, birth and death rates, and population size. Furthermore, our results explain why the deviation of the mutant allele frequency from the 1/*f* power law in an intermediate frequency range is not a sensitive statistic for detecting subclonal selection in models of exponentially growing cancer populations: mutational frequency distribution of a subclonal driver is convex and thus always biased toward 0% or 100% frequency.

In this paper, we study a process in which we explicitly include selection, and show that a subclonal driver, though present, may often fail to change the VAF distribution expected from neutral evolution. We note that, if there is no subclonal driver present, i.e. when driver mutation frequency is precisely *f* = 0 or *f* = 1, the neutral test developed by Williams et al. [13] will be correct. However, due to the finite resolution with which we can distinguish different mutation frequencies using current sequencing techniques, it will be difficult if not impossible to determine that a mutation is present at precisely 0 or 1 frequency (e.g. experimental *f* = 0.9 may correspond to *f* = 1 due to sequencing errors and vice versa; low frequency mutations may have experimental *f* = 0 due to insufficient sequencing depth).

Our conclusions do not contradict the results of many recent genomic studies that find large subclones in majority of sequenced cancers [29–31]; many of these subclones may in fact be lacking functional driver mutations and/or can be a consequence of genetic drift. For example, a recent genomic study of chronic lymphocytic leukemia [30] (CLL) reports that the majority of macroscopic (>10% cancer cell frequency) CLL subclones seem to be passenger subclones that lack selective advantage over their parent subclones. Our results are in agreement with the recent Williams et al. study [15], which found that 21% of colon cancers, 29% of gastric cancers and 53% of metastases they examined had evidence of differentially selected (driver) subclones. These estimates are in line with our predictions of how likely it would be to detect selection even if 100% of tumors were non-neutral.

On the other hand, Nik-Zainal et. al. [32] find dominant subclones (>50% CCF) in all 21 breast tumors they studied, and argue that these dominant subclones are likely to have been selected (i.e. that they are driver subclones). We argue that detecting subclonal drivers is in general challenging; if all dominant subclones from Nik-Zainal et al. [32] did in fact contain drivers, it may mean that these tumors have evolved differently from the model and parameters we have assumed in this paper. Yates et al. [31] find subclonal driver mutations in 15/50 (30%) of breast cancers they studied; Turajlic et al. [33] found subclonal driver mutations in 120/216 (56%) of primary and metastatic renal cancers they sequenced. The fractions of samples with detectable subclonal driver mutations in these two studies are in line with the average driver detection probability in our model with two drivers, which is 43%.

The two-type model we have studied here is a simplification of the process of driver accumulation in tumors. Our model is deliberately oversimplified to allow for analytic treatment and to develop an intuition into why subclonal drivers may elude detection. In reality, detecting subclonal drivers will be even more difficult due to experimental uncertainties, confounding passenger mutations, and a contribution from contaminating non-cancer cells. For example, we and others [13,15] implicitly assume that genomic analysis of biopsy samples identifies true cancer cell frequencies of mutations in the entire tumor population. However, significant spatial heterogeneity may exist in solid tumors, and a minor subclone may be dominant in certain spatial regions of the tumor and overrepresented in a tumor biopsy, resulting in possible misleading conclusions derived from the mutational frequency spectrum of an unrepresentative sample [34].

There is a debate about mini-drivers in cancer [35], referring to mutations that are in between strong and/or very frequently mutated drivers on the one hand, and neutral passenger mutations on the other. Our study demonstrates that strong drivers (growth rate increase g = 70%) are most likely to be detected from macroscopic subclonal frequencies for moderately-growing tumors and a typical driver mutation rate (*u*~10^−5^). For higher driver mutation rates and/or slower-growing tumors, growth advantage of most likely detectable drivers decreases. In particular, our results demonstrate that mini-drivers will most likely be detectable in very slow-growing tumors, that are already close to or at carrying capacity.

Finally, our conclusions are relevant not only to cancer but more generally to the problem of measuring selection when an expanding subpopulation of fitter cells coexist with “wild-type” cells, such as growing bacterial populations acquiring *de novo* resistance to antibiotics or adapting to a new environment.

## Materials and methods

### Frequency distribution of a driver subclone

We study a two-type continuous time branching process that starts with a single type-0 cell. With rate *b*, type-0 cells divide into two identical daughter cells. Death rate of type-0 cells is *d*, with *b* > *d*. In addition, type-0 cells can receive an additional driver mutation with rate *u*. We will assume that the driver mutation rate is very small, on the order of *u* ~ 10^−5^ (per day). Cells with the additional driver divide with rate *b*_1_ and die with rate *d*_1_, again with *b*_1_ > *d*_1_. The net growth rate of cells with the additional driver, *r*_1_ = *b*_1_ − *d*_1_, is greater than the net growth rate of type-0 cells, *r* = *b* − *d*. We will denote the ratio of the two growth rates *r*_1_ and *r* by *c* = *r*_1_/*r* > 1. Let *X*_0_ be the number of type-0 cells at the appearance of the first successful cell with a driver (whose progeny survives stochastic fluctuations). The progeny of this cell forms the type-1 population. Total population size is the sum of type-0 and type-1 cells.

We are interested in the probability distribution of the fraction *f*_sub_ of type-1 cells in the population when total population size is *M*. Typical size *M* that we will consider is 10^8^ − 10^9^ cells. If we let *X* be the size of the type-0 population and *Y* the size of the type-1 population when total population size is *M*, then *f*_*sub*_ = *Y*/*M* and *M* = *X*+*Y*.

Survival probability of a cell with the additional driver mutation is *r*_1_/*b*_1_ = *cr*/*b*_1_. Thus the "successful" driver mutation rate (the rate at which driver cells with surviving progeny are produced) is *u*_s_ = (*cr*/*b*_1_)*u*. On the other hand, we have shown before that the arrival of mutations which appear with rate us in type-0 cells, can be viewed as a Poisson process with rate *u*_s_/*r* on the size of the type-0 population [37]. More precisely, we use the fact that the number of mutations produced by the type-0 population by the time it reaches population size *M* is distributed as Poisson(*M u*_s_ /*r*). This result was derived using heuristic arguments by Iwasa et al. [38] and Bozic and Nowak [37], and proved recently by Cheek and Antal [18]. The size of the type-0 population when the first type-1 cell appears, *X*_0_, is therefore exponentially distributed with rate (*c*/*b*_1_)*u*. Thus *X*_0_ will be of the order of *b*_1_/(*cu*), which is typically much larger than 1, but much smaller than *M*. We note that, in line with multiple studies of similar branching processes [39,37], we use a continuous approximation to the size of the type-0 population at the time of mutant appearance. As typical driver mutation rate is *u*~10^−5^, driver mutation is expected to appear when type-0 population contains ~10^5^ cells, so this approximation is justified.

We will measure time from the appearance of the first type-1 cell. Let *t* be the time when total population size is *M*. Since *X*_0_ is typically very large, the population of type-0 cells at time *t* can be well approximated by *X* ≈ *e*^*rt*^ *X*_0_. On the other hand, since type-1 cells started a surviving population at time 0 with a single cell, for the population of type-1 cells we have [39] *Y* → *V*_1_*e*^*crt*^ for large *t*, where *V*_1_ is an exponentially distributed random variable with rate *cr*/*b*_1_. In other words, *Y* ≈ *V*_1_ (*X* / *X*_0_)^c^. It follows that

$$M=X+Y\approx X+V_{1}{\left(X/X_{0}\right)}^{c}$$

$$M^{1-c}\approx (X/M)M^{1-c}+V_{1}{\left(X/M\right)}^{c}X_{0}^{-c}$$

$$1\approx (1-f_{sub})+V_{1}{\left(1-f_{sub}\right)}^{c}X_{0}^{-c}M^{c-1}$$

$$\frac{f_{sub}}{{\left(1-f_{sub}\right)}^{c}}\approx V_{1}X_{0}^{-c}M^{c-1}$$ (3)

Here we used the fact that *X* / *M* = 1 − *f*_*sub*_. On the other hand,

$$P[f_{sub}\leq \alpha ]=P[\frac{f_{sub}}{{\left(1-f_{sub}\right)}^{c}}\leq \frac{\alpha }{{\left(1-\alpha \right)}^{c}}],$$ (4)

since *x*/(1-*x*)^c^ is a function that increases as *x* increases from 0 to 1. Thus from (3) and (4) we have

$$\begin{array}{ll} P\left[f_{sub}\leq \alpha \text{|}X_{0}\right] & \approx P\left[V_{1}X_{0}^{-c}M^{c-1}\leq \frac{\alpha }{{\left(1-\alpha \right)}^{c}}\right]\\ & =P\left[V_{1}\leq \frac{\alpha }{{\left(1-\alpha \right)}^{c}}X_{0}^{c}M^{1-c}\right]\\ & =1-\text{exp}\left(-\left(cr/b_{1}\right)\frac{\alpha }{{\left(1-\alpha \right)}^{c}}X_{0}^{c}M^{1-c}\right) \end{array}$$ (5)

Finally we have

$$P[f_{sub}\leq \alpha ]\approx \int_{0}^{M}\frac{cu}{b_{1}}exp(-\frac{cu}{b_{1}}X_{0})[1-exp(-\frac{cr}{b_{1}}{\frac{\alpha }{{\left(1-\alpha \right)}^{c}}X}_{0}^{c}M^{1-c})]dX_{0}$$ (6)

**Fig 4:**
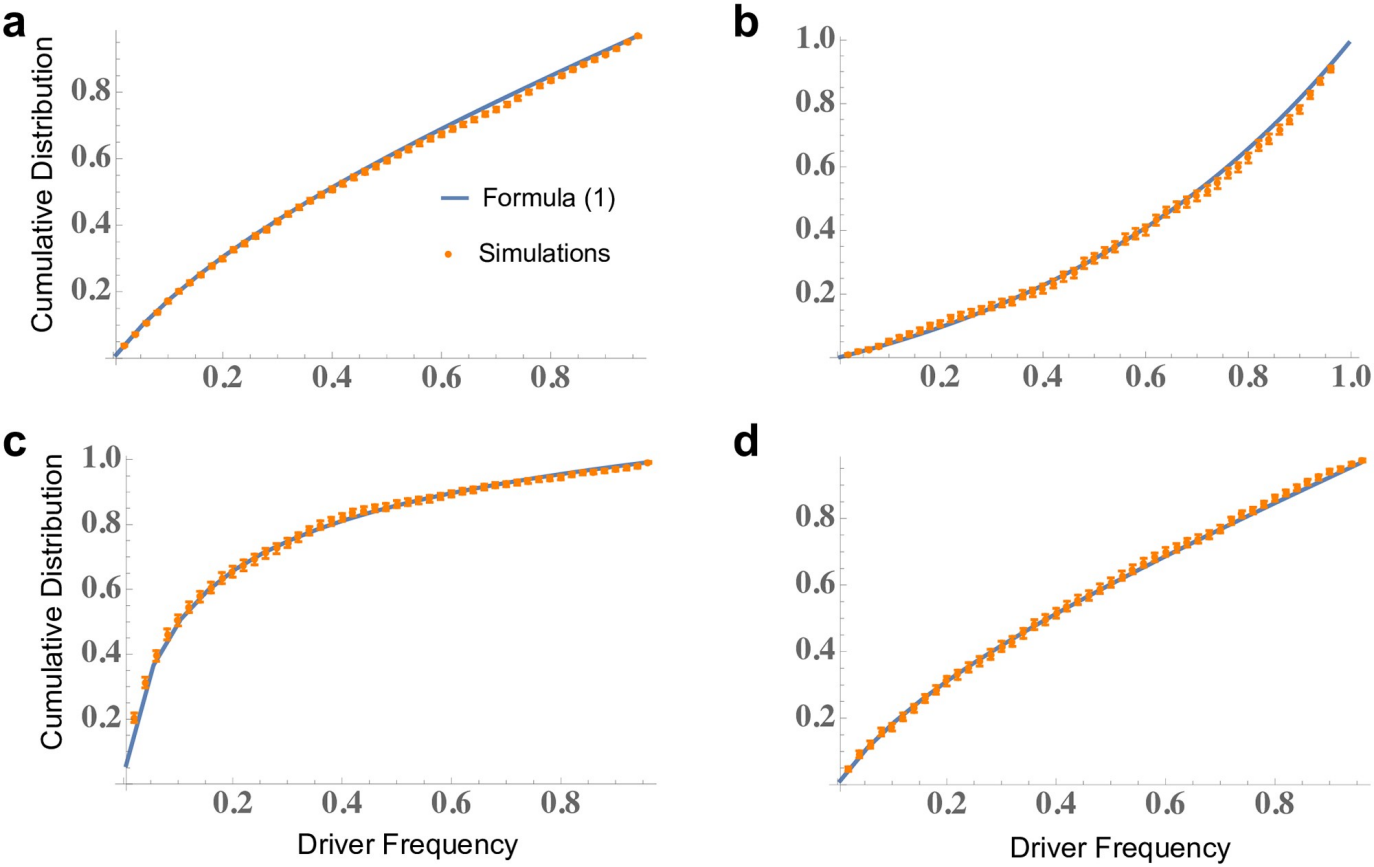
Comparison of formula (1) for the cumulative distribution of driver frequency and exact computer simulations. On the y-axis we plot the probability that driver frequency is below a particular value. Error bars are standard errors of the mean (s.e.m.) obtained via bootstrapping. Parameters: **a**, *b* = *b*_1_ = 0.14, *d* = 0.13, *c* = 1.7, *u* = 10^−5^, *M* = 10^8^; **b**, *b* = *b*_1_ = 0.14, *d* = 0.13, *c* = 1.5, *u* = 10^−4^, *M* = 10^8^; **c**, *b* = *b*_1_ = 0.14, *d* = 0.13, *c* = 1.5, *u* = 10^−5^, *M* = 10^8^; **d**, *b* = *b*_1_ = 0.14, *d* = 0.17, *c* = 1.9, *u* = 10^−5^, *M* = 10^8^.

We show the excellent agreement of formula (6) and exact computer simulations of the process in Fig 4.

Let

$$H(\alpha )=\frac{cu}{b_{1}}exp(-\frac{cu}{b_{1}}X_{0})[1-exp(-\frac{cr}{b_{1}}{\frac{\alpha }{{\left(1-\alpha \right)}^{c}}X}_{0}^{c}M^{1-c})]$$

To calculate the probability density function, *f*, for the frequency of subclonal driver, we note that

$$f(\alpha )=\frac{d}{d\alpha }P[f_{sub}\leq \alpha ]=\frac{d}{d\alpha }\int_{0}^{M}H(\alpha )dX_{0}$$

Using Leibniz’s rule we obtain

$$f(\alpha )=\int_{0}^{M}\frac{d}{d\alpha }H(\alpha )dX_{0}$$

Finally, probability density function for the frequency of a subclonal driver is given by

$$f(\alpha )={\left(\frac{c}{b_{1}}\right)}^{2}ruM^{1-c}{\left(1-\alpha \right)}^{-1-c}(1+(-1+c)\alpha )\int_{0}^{M}X_{0}^{c}\;\text{exp}(-\frac{c}{b_{1}}(uX_{0}+rM^{1-c}X_{0}^{c}{\left(1-\alpha \right)}^{-c}\alpha ))dX_{0}$$ (7)

### Estimating driver mutation rate

The estimated driver mutation rate *u* ∼ 10^−5^ used in Fig 2 comes from Bozic et al. [21]. In that paper, it was estimated that there are 377 driver genes in the human genome, and an average of 90 positions per driver gene that, if mutated, will result in a functional driver mutation. In addition, it was assumed that the mutation rate per base pair per cell division was 5 · 10^−10^, leading to a driver mutation rate on the order of 10^−5^ per cell division.

Since then, new estimates have become available for both the number of driver genes and the point mutation rate in tumors. For example, Vogelstein et al. [9] used mutation patterns to estimate that there are 138 driver genes discovered so far. Similarly, Davoli et al. [10] analyzed patterns of mutational signatures in tumors and estimated 570 driver genes. Lawrence et al. [12] used mutation frequencies and estimated 219 driver genes. They also performed a saturation analysis and showed that many new candidate cancer genes remain to be discovered beyond those they report. Recently, Martincorena et al. [11] used dN/dS ratio to determine genes under positive selection in cancer and estimated 203 driver genes. Based on the sum of these data, we set the upper bound on the number of driver genes to be 600.

On the other hand, if we only focus on strong drivers in a single cancer type, such as colorectal, the number of genes is significantly smaller. For example, Martincorena et al. [11] report 28 genes under significant positive selection in colorectal cancer. Thus we will set the lower bound on the number of significant driver genes of a single cancer type to 20.

Blokzijl et al. [40] estimate that ∼ 40 mutations accumulate per year in the genome of multiple human tissues, including the small intestine, colon and liver, leading to a mutation rate of 0.1/day per genome or ∼ 4 · 10^−11^ per base pair per day. This will be our lower bound for the point mutation rate. Recently, Werner and Sottoriva [41] used the change in the mean and variance of the mutational burden with age in healthy human tissues to estimate the mutation rate in the colon and small intestine; they obtained ∼ 4 * 10^−9^ per base pair per cell division. Assuming the value they used for time between stem cell divisions is one week, this leads to a mutation rate of ∼ 6 * 10^−10^ per base pair per day. Mutation rate in cancer can be increased 10–100 fold compared to normal tissues [42], so we set the upper bound for point mutation rate to ∼ 100*6·10^−10^ = 6·10^−8^ per base pair per day.

We obtain an upper bound for the driver mutation rate by multiplying the upper bounds for the number of driver genes and point mutation rate with the average number of driver positions, leading to *u*_U_ = 600*6·10^−8^ *90∼10^−3^ per day.

Multiplying our lower bounds for the number of driver genes and point mutation rate with the average number of driver positions leads to the lower bound for the driver mutation rate *u*_L_ = 20*4·10^−11^ *90∼10^−7^.

### Subclonal driver frequency is biased towards 0 and 1 over a large range of parameter values

Using formula (6), we numerically evaluate *P* [0.2 < *f*_sub_ ≤ 0.8] = *F* (0.8) − *F* (0.2) for the following ranges of parameters: ratio of net growth rates of cells with and without the driver, *c*, between 1.01 and 3 (i.e. relative growth rate advantage of a driver, *g*, between 1% and 200%); driver mutation rate, *u*, between 10^−7^ and 10^−3^ per day; final tumor size, *M*, between 10^7^ and 10^11^ cells; division rate of cells with the driver, *b*_1_, between 0.1 and 1 per day; and net growth rate of tumor cells, *r*, between 0.001 and 0.1 per day. These ranges are wide and include all biologically meaningful parameter values.

We create a grid by taking 100 equally-spaced values for each parameter within its range defined above (parameter values for driver mutation rate *u*, tumor size *M* and net growth rate of tumor cells *r* are equally spaced in log space). We evaluate all points on this 5-dimensional grid (100^5^ = 10^10^ parameter value combinations). We find that *P* [0.2 < *f*_sub_ ≤ 0.8] < 0.6 holds everywhere, and that the frequency of a subclonal driver is always biased toward 0 and 1.

### Computer simulations

#### Well mixed model

We perform Monte Carlo simulations of the branching process model using the Gillespie algorithm [43]. We start the simulation with a single type-0 cell. At each iteration, time to next event and the type of event are drawn randomly depending on the current composition of the population. In particular, cells can die with rate *d* (type-0 cells) or *d*_1_ (type-1), divide into two cells with rate *b* (type-0) or *b*_1_ (type-1), or a type-0 cell mutates to type-1 cell with rate *u*. In the simulation, we keep track of the numbers of type-0 cells and of multiple type-1 populations, until the first type-1 population that is certain to survive stochastic drift appears, at which point we only keep track of that type-1 population. We stop the simulation once the total population (sum of type-0 and type-1 cells) reaches final size *M*. We perform between 1000 and 5000 surviving runs for each parameter combination.

#### Spatial model

We use the model from our previous work [5] but without migration. Cells occupy points on a 3d lattice. Each simulation starts from a single cell of type 0. At each time step, a cell either dies with rate *d* (type 0 cell) or *d*_1_ (type 1 cell), or replicates with rate *b* times the fraction of empty neighbors. A cell that is completely surrounded by other cells does not replicate. Replication can generate a mutant (type 1 cell) with probability *u*/*b* (equivalent to the rate of mutant generation being *u*), where *u* is the driver mutation rate as in the well-mixed model. Once a mutant has been generated, further mutations are forbidden. This is to ensure that only a single driver is present. Simulations that do not produce a driver (unlikely) or where the population goes extinct due to a stochastic fluctuation are rejected. The simulation is stopped when the tumor reaches a given final size *M*. The fraction of cells with the driver mutation (type 1 cells) is determined, and the simulation is repeated 1000 times to obtain the experimental driver frequency distribution. The list of driver frequencies is also used as input for the combined 3d-logistic growth model.

## Supporting information

S1 Fig Probability of detection of a subclonal driver in a 3d spatial model. Probability that a subclonal driver is in the detectable range (0.2 ≤ *f*_*sub*_ ≤ 0.8) for three parameter regimes (medium, fast and slow-growing tumor). For each parameter regime, we depict three levels of selection: moderate selection (driver increases net growth rate *b* − *d* by *g* = 30%), strong selection (*g* = 70%), and very strong selection (*g* = 100%). Birth rate of all cells [5] is *b* = *b*_1_ = 1 (see Materials and methods for details of the simulation). Death rate of cells without the driver [5] for **a**, moderately growing tumor: *d* = 0.7; **b**, fast growing tumor: *d* = 0.5; **c**, slow-growing tumor: *d* = 0.9. Driver mutation rate *u* = 10^−5^. All rates are per day. Error bars are s.e.m.
(TIF)

S2 Fig Probability of detection of a subclonal driver in a sigmoidal model. Probability that a subclonal driver is in the detectable range (0.2 ≤ *f*_*sub*_ ≤ 0.8) for three parameter regimes (medium, fast and slow-growing tumor). For each parameter regime, we depict three levels of selection: moderate selection (driver increases initial growth rate *r* by *g* = 30%), strong selection (*g* = 70%), and very strong selection (*g* = 100%). Parameter values for **a**, moderately growing tumor: *r* = 0.01; **b**, fast growing tumor: *r* = 0.07; **c**, slow-growing tumor: *r* = 0.0013. Carrying capacity *K* = 10^11^ cells. Error bars are s.e.m.
(TIF)

S3 Fig Probability of detection of a subclonal driver in model with two sequential drivers. Probability that a subclonal driver is in the detectable range (0.2 ≤ *f*_*sub*_ ≤ 0.8) for three parameter regimes. Orange line denotes the probability of detection of first driver, and blue line represents probability of detection of second driver assuming that first driver is undetectable. Red line depicts the probability of detection of any driver (orange + blue). For each parameter regime, we depict three levels of selection: moderate selection (first driver increases net growth rate *r* by *g* = 30%, left), strong selection (*g* = 70%, middle), and very strong selection (*g* = 100%, right). Second drivers increase the net growth rate by the same absolute amount (*gr*). Parameter values for **a**, moderately growing tumor: *b* = 0.14, *r* = 0.01; **b**, fast growing tumor: *b* = 0.25, *r* = 0.07; **c**, slow-growing tumor: *b* = 0.33, *r* = 0.0013. Driver mutation rate *u* = 10^−5^. All rates are per day and *b* = *b*_1_.
(TIF)
