# Supplementary figures and images for: On measuring selection in cancer from subclonal mutation frequencies
Source: PLoS Comput Biol. 2019 Sep 26;15(9):e1007368. doi: 10.1371/journal.pcbi.1007368 (PMC6788714; doi:10.1371/journal.pcbi.1007368)

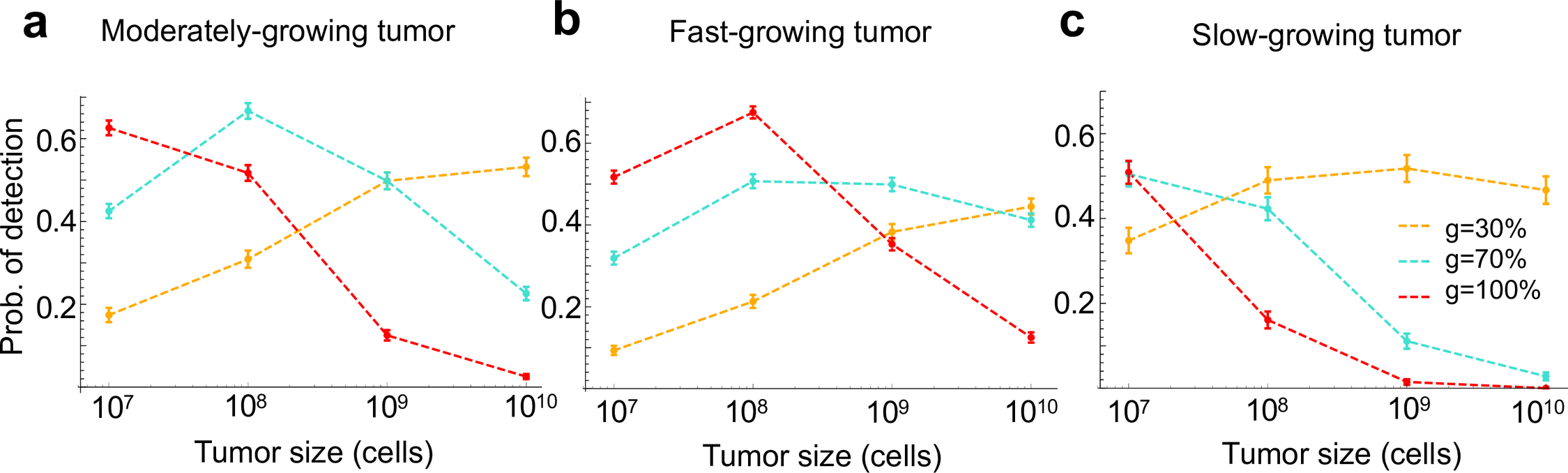

Supplement: S1 Fig — Probability that a subclonal driver is in the detectable range (0.2 ≤ fsub ≤ 0.8) for three parameter regimes (medium, fast and slow-growing tumor). For each parameter regime, we depict three levels of selection: moderate selection (driver increases net growth rate b − d by g = 30%), strong selection (g = 70%), and very strong selection (g = 100%). Birth rate of all cells [5] is b = b1 = 1 (see Materials and methods for details of the simulation). Death rate of cells without the driver [5] for a, moderately growing tumor: d = 0.7; b, fast growing tumor: d = 0.5; c, slow-growing tumor: d = 0.9. Driver mutation rate u = 10−5. All rates are per day. Error bars are s.e.m. (TIF) [file pcbi.1007368.s001.tif]

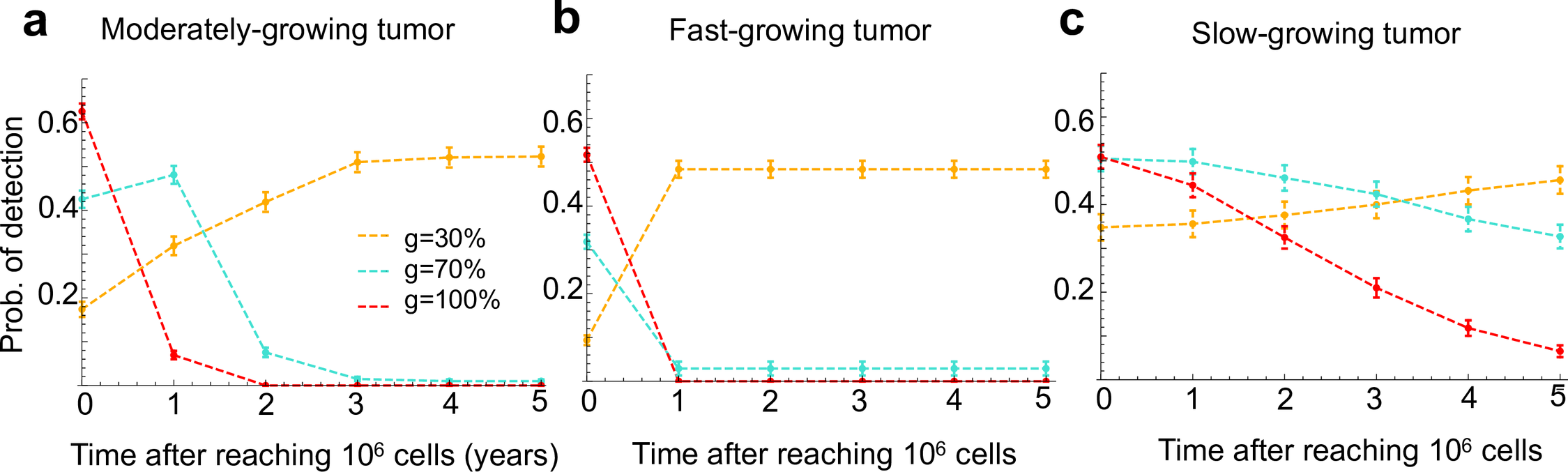

Supplement: S2 Fig — Probability that a subclonal driver is in the detectable range (0.2 ≤ fsub ≤ 0.8) for three parameter regimes (medium, fast and slow-growing tumor). For each parameter regime, we depict three levels of selection: moderate selection (driver increases initial growth rate r by g = 30%), strong selection (g = 70%), and very strong selection (g = 100%). Parameter values for a, moderately growing tumor: r = 0.01; b, fast growing tumor: r = 0.07; c, slow-growing tumor: r = 0.0013. Carrying capacity K = 1011 cells. Error bars are s.e.m. (TIF) [file pcbi.1007368.s002.tif]

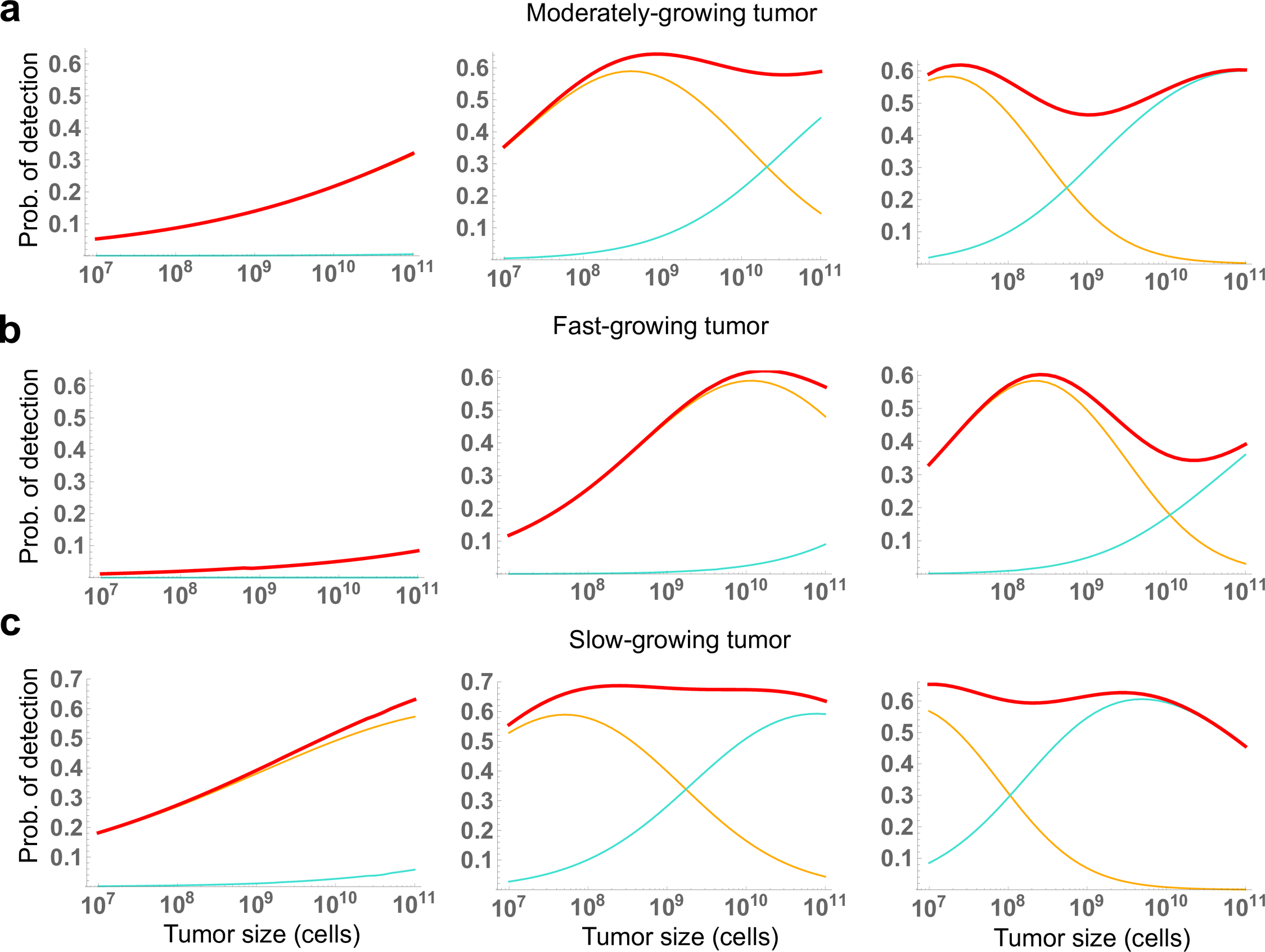

Supplement: S3 Fig — Probability that a subclonal driver is in the detectable range (0.2 ≤ fsub ≤ 0.8) for three parameter regimes. Orange line denotes the probability of detection of first driver, and blue line represents probability of detection of second driver assuming that first driver is undetectable. Red line depicts the probability of detection of any driver (orange + blue). For each parameter regime, we depict three levels of selection: moderate selection (first driver increases net growth rate r by g = 30%, left), strong selection (g = 70%, middle), and very strong selection (g = 100%, right). Second drivers increase the net growth rate by the same absolute amount (gr). Parameter values for a, moderately growing tumor: b = 0.14, r = 0.01; b, fast growing tumor: b = 0.25, r = 0.07; c, slow-growing tumor: b = 0.33, r = 0.0013. Driver mutation rate u = 10−5. All rates are per day and b = b1. (TIF) [file pcbi.1007368.s003.tif]
